# Validation of a new WIND classification compared to ICC classification for weaning outcome

**DOI:** 10.1186/s13613-018-0461-z

**Published:** 2018-11-29

**Authors:** Byeong-Ho Jeong, Kyeong Yoon Lee, Jimyoung Nam, Myeong Gyun Ko, Soo Jin Na, Gee Young Suh, Kyeongman Jeon

**Affiliations:** 1 Division of Pulmonary and Critical Care Medicine, Department of Medicine, Samsung Medical Center, Sungkyunkwan University School of Medicine, 81 Irwon-ro, Gangnam-gu, Seoul, 06351 Republic of Korea; 2 Intensive Care Unit Nursing Department, Samsung Medical Center, Sungkyunkwan University School of Medicine, Seoul, Republic of Korea; 3 Department of Critical Care Medicine, Samsung Medical Center, Sungkyunkwan University School of Medicine, Seoul, Republic of Korea

**Keywords:** Mechanical ventilation, Ventilator weaning, Treatment outcome, Classification

## Abstract

**Background:**

Although the WIND (Weaning according to a New Definition) classification based on duration of ventilation after the first separation attempt has been proposed, this new classification has not been tested in clinical practice. The objective of this cohort study was to evaluate the clinical relevance of WIND classification and its association with hospital mortality compared to the International Consensus Conference (ICC) classification.

**Methods:**

All consecutive medical ICU patients who were mechanically ventilated for more than 24 h between July 2010 and September 2013 were prospectively registered. Patients were classified into simple, difficult, or prolonged weaning group according to ICC classification and Groups 1, 2, 3, or no weaning (NW) according to WIND classification.

**Results:**

During the study period, a total of 1600 patients were eligible. These patients were classified by the WIND classification as follows: Group NW = 580 (36.3%), Group 1 = 617 (38.6%), Group 2 = 186 (11.6%), and Group 3 = 217 (13.6%). However, only 735 (45.9%) patients were classified by ICC classification as follows: simple weaning = 503 (68.4%), difficult weaning = 145 (19.7%), and prolonged weaning = 87 (11.8%). Clinical outcomes were significantly different across weaning groups by ICC classification and WIND classification. However, there were no statistical differences in successful weaning rate (96.6% vs. 95.2%) or hospital mortality (22.5% vs. 25.5%) between simple and difficult weaning groups by the ICC. Conversely, there were statistically significant differences in successful weaning rate (98.5% vs. 76.9%) and hospital mortality (21.2% vs. 33.9%) between Group 1 and Group 2 by WIND.

**Conclusions:**

The WIND classification could be a better tool for predicting weaning outcomes than the ICC classification.

**Electronic supplementary material:**

The online version of this article (10.1186/s13613-018-0461-z) contains supplementary material, which is available to authorized users.

## Introduction

Weaning from mechanical ventilation (MV) is a complex process involving daily assessment of readiness to wean and spontaneous breathing trial (SBT) to extubation [1]. The weaning process comprises at least 40% of the total duration of MV [2], and prolonged weaning is associated with higher mortality [3, 4]. A good understanding of the weaning process will reduce the duration of MV, lead to successful extubation, and eventually reduce the mortality rate and length of stay (LOS) in the intensive care unit (ICU) [1, 5].

In 2007, an International Consensus Conference (ICC) on weaning from MV proposed a classification into three different groups (simple, difficult, and prolonged weaning) according to the number, duration, and results of SBTs as well as extubation outcomes to simply classify and deeply understand the weaning process [1]. However, ICC classification had some problems when applied in clinical practice: (a) it does not apply to patients without a weaning trial (unplanned extubation, death, or transfer out), (b) patients with tracheostomy tube before weaning trials are difficult to classify with ICC, and (c) ICC classification is based only on the successful results of SBT. Therefore, approximately half of mechanically ventilated patients could not be classified by the ICC classification [3, 4, 6, 7]. To overcome these limitations, the WIND (Weaning according to a New Definition) Study Group and the REVA (Réseau Européen de Recherche en Ventilation Artificielle) Network proposed a new classification using four different groups (Groups 1, 2, 3, and no weaning [NW]) [8]. However, WIND classification has not yet been fully validated and has not been sufficiently compared with ICC classification. Therefore, the objective of this cohort study was to evaluate the clinical relevance of WIND classification and its association with hospital mortality compared to ICC classification.

## Methods

### Study population

All consecutive patients admitted to the medical ICU and requiring MV for more than 24 h between July 2010 and September 2013 were prospectively registered at Samsung Medical Center, a 1989-bed tertiary referral hospital with tertiary-level ICU, in Seoul, South Korea [3, 9, 10]. If a patient was re-admitted to the ICU for MV support during the same hospital admission, only the first weaning episode was included in analysis. Multiple ICU visits during different hospital admissions were enrolled separately. Patients who were transferred from other hospitals after more than 48 h of intubation or were successfully treated by noninvasive ventilation (NIV) were excluded. The Institutional Review Board of Samsung Medical Center approved this study and allowed review and publication of information from patient records. Informed consent was waived because of the study’s observational nature.

### Standardized weaning process

Since 2010, the medical ICU of our hospital has utilized a specific protocol-based weaning program according to the recommendations by Boles et al. [1]. Details of our weaning program were described in previous reports [3, 9, 10] and an additional file provided. In short, respiratory care practitioners (RCP), who are registered nurses specializing in respiratory care, screened patients daily for weaning readiness and conducted SBTs according to the protocol. When a patient passed the SBT, extubation proceeded. If a patient failed the SBT, MV was resumed, and the team reviewed possible reversible etiologies for the failure. Again, when a patient proved ready for weaning, the SBT was repeated the following day.

### Weaning classification by ICC and WIND

**Fig. 1:**
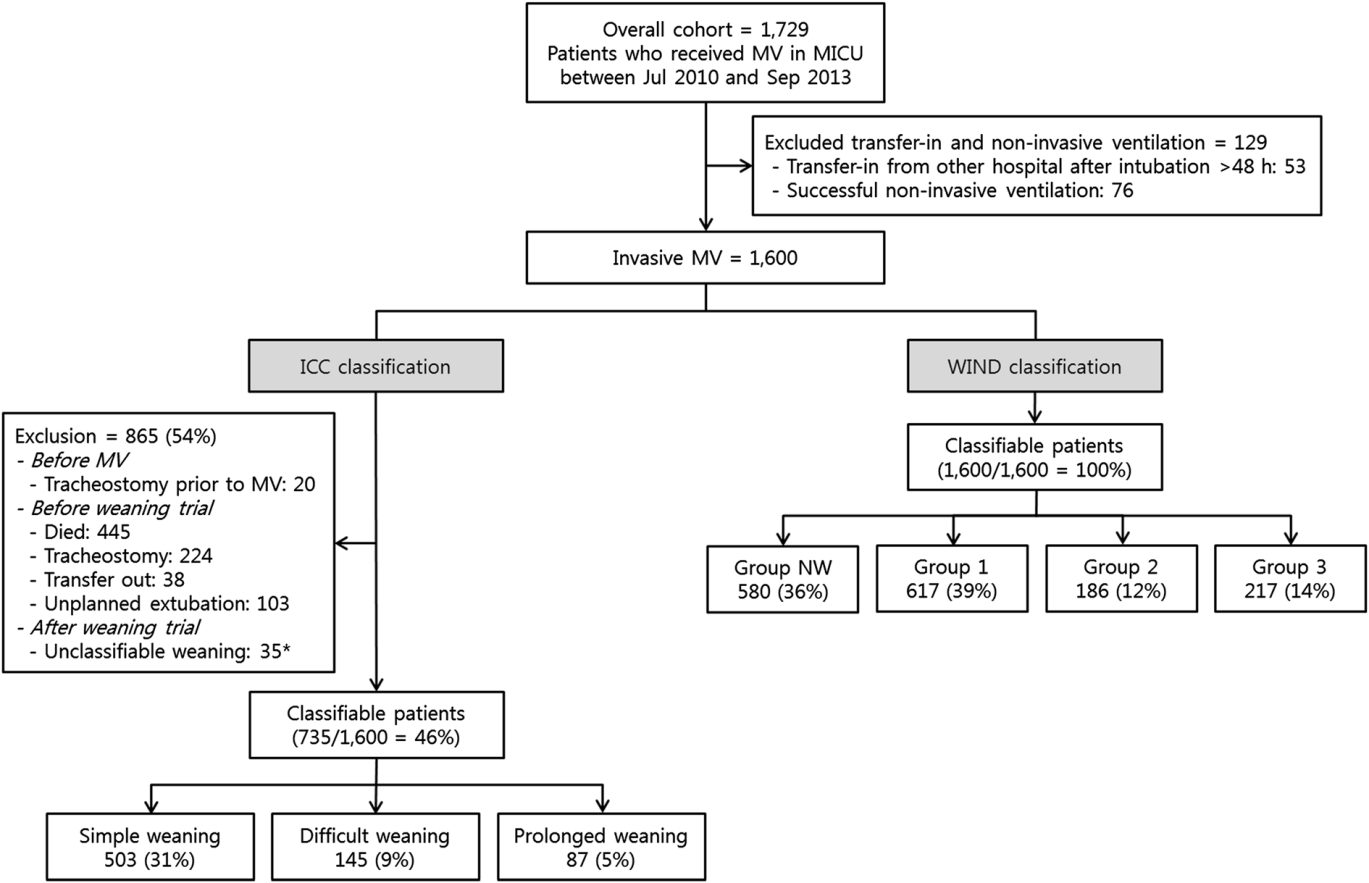
Flowchart of WIND and ICC classifications. *Patients who died or were transferred to other hospitals after failure of first SBT and before third SBT or 7 days. *MV* mechanical ventilation, *MICU* medical intensive care unit, *ICC* International Consensus Conference, *WIND* Weaning according to a New Definition, *NW* no weaning

Patients were classified into simple, difficult, or prolonged weaning groups according to ICC classification [1] and Groups 1, 2, 3, or NW according to WIND classification [8]. The three weaning groups by ICC classification were defined as follows: simple weaning, patients who proceed from initiation of weaning to successful extubation (no need to reinstitute ventilator support within 48 h of extubation) on the first attempt without difficulty; difficult weaning, patients who failed initial weaning and required up to three SBTs or as long as 7 days from the first SBT to achieve successful extubation; or prolonged weaning, patients who required more than three SBTs or > 7 days of weaning after the first SBT. To apply the ICC classification, unclassifiable patients were excluded as follows: patients with tracheostomy prior to MV; patients who died, underwent tracheostomy, transferred out, or had unplanned extubation before weaning trial; and patients with unclassifiable weaning after SBT who died or were transferred to another hospital after failure of the first SBT and before the third SBT or 7 days (Fig. 1). The four weaning groups by WIND classification were defined as follows: Group NW, patients who never experienced any separation attempt (SA); Group 1, the first SA resulted in termination of the weaning process within 1 day (successful separation or early death); Group 2, weaning was completed after more than 1 day but in less than 1 week after the first SA (successful separation or death); and Group 3, weaning was not terminated by 7 days after the first SA (by successful separation or death). In WIND classification, SA is defined as SBT or extubation directly performed without SBT (including unplanned extubation) for intubated patients and as ≥ 24 h with spontaneous ventilation through tracheostomy without any mechanical ventilation for tracheostomized patients.

### Weaning outcomes

To analyze differences in weaning outcomes among groups according to ICC and WIND classifications, clinical outcomes of MV days, ventilator-free days, tracheostomy rate, successful weaning rate, ICU mortality, LOS in ICU, hospital mortality, and LOS in hospital were investigated. Ventilator-free days were calculated as the number of days without invasive ventilation to day 28. Nonsurvivors were considered as patients with 0 ventilator-free days. Because there is no applicable definition for tracheostomized patients in ICC classification, successful weaning was defined according to WIND definitions as follows: for intubated patients, extubation without death or reintubation within the next 7 days whether postextubation NIV was used or not or ICU discharge without invasive MV within 7 days, whichever comes first; for tracheostomized patients, spontaneous ventilation through tracheostomy without any MV for 7 consecutive days or discharged with spontaneous breathing, whichever comes first. The date of successful weaning was counted to the actual day of extubation or spontaneous ventilation through tracheostomy after the patient had completed 7 days without reintubation or any MV through tracheostomy (or was alive and discharged earlier).

### Statistical analysis

The data are presented as medians and interquartile ranges (IQR) for continuous variables and as numbers and percentages for categorical variables. The Jonckheere–Terpstra test for continuous variables [11] and the Mantel–Haenszel test for categorical variables [12] were used to analyze trends of baseline characteristics and outcomes across weaning groups. The Mann–Whitney *U* test was used for continuous variables, and Pearson’s Chi-square test was used for categorical variables to identify statistical differences of main weaning outcomes between weaning groups according to the ICC and WIND classifications, respectively. All tests were two-tailed, and a *P* value < 0.05 was considered significant. The data were analyzed using PASW Statistics 18 (SPSS Inc., Chicago, IL, USA).

## Results

### Application of ICC and WIND classifications to the same cohort

During the study period, a total of 1600 patients were eligible after excluding patients who transferred from other hospitals after more than 48 h of intubation (*n* = 53) or underwent successful NIV (*n* = 76) (Fig. 1). All eligible patients were classified by the WIND classification as follows: Group NW = 580 (36.3%), Group 1 = 617 (38.6%), Group 2 = 186 (11.6%), and Group 3 = 217 (13.6%). However, only 735 (45.9%) patients could be classified by the ICC classification as follows: simple weaning = 503 (31.4%), difficult weaning = 145 (9.1%), and prolonged weaning = 87 (5.4%).

Baseline characteristics of the total cohort are presented in Table 1. Median age was 65 years, and 68.0% of patients were male. The most common comorbidity was malignant disease (59.5%), and the most common cause of respiratory failure was pneumonia (33.4%), followed by extrapulmonary sepsis (21.6%) and acute respiratory distress syndrome (9.8%).

**Table 1:** Baseline characteristics of the total cohort, excluded patients by ICC classification, and Group NW by WIND classification

Variables	Total (*n* = 1600)	ICC	WIND
		Excluded (*n* = 865)	Group NW (*n* = 580)
Age, years	65 (54–72)	63 (54–72)	63 (52–71)
Sex, male	1088 (68.0)	580 (67.1)	387 (66.7)
Underlying disease			
Malignancy	952 (59.5)	561 (64.9)	389 (67.1)
Respiratory	458 (28.6)	250 (28.9)	156 (26.9)
Neurologic	225 (14.1)	122 (14.1)	60 (10.3)
Gastrointestinal	209 (13.1)	135 (15.6)	108 (18.6)
Cardiovascular	202 (12.6)	99 (11.4)	65 (11.2)
Genitourinary	171 (10.7)	70 (8.1)	48 (8.3)
Interval between hospital admission and ICU admission, days	2 (0–11)	3 (0–14)	3 (0–14)
Cause of respiratory failure			
Pneumonia	535 (33.4)	270 (31.2)	165 (28.4)
Extrapulmonary sepsis	345 (21.6)	201 (23.2)	165 (28.4)
ARDS	157 (9.8)	93 (10.8)	66 (11.4)
CPR	114 (7.1)	73 (8.4)	55 (9.5)
Coma	88 (5.5)	51 (5.9)	29 (5.0)
Pulmonary edema	76 (4.8)	20 (2.3)	15 (2.6)
Ventilatory failure	63 (3.9)	24 (2.8)	12 (2.1)
Central airway obstruction	55 (3.4)	36 (4.2)	11 (1.9)
Exacerbation of ILD	35 (2.2)	18 (2.1)	14 (2.4)
Others	132 (8.3)	79 (9.1)	48 (8.3)
SAPS III on ICU admission	64 (53–77)	66 (55–79)	68 (58–83)
SOFA score on ICU admission	9 (6–13)	11 (8–14)	12 (8–15)
Interval between hospital admission and intubation, days	2 (0–12)	4 (0–16)	4 (0–16)

*ICC* International Consensus Conference, *NW* no weaning, *WIND* Weaning according to a New Definition, *ICU* intensive care unit, *ARDS* acute respiratory distress syndrome, *ILD* interstitial lung disease, *CPR* cardiopulmonary resuscitation, *SAPS III* Simplified Acute Physiology Score III, *SOFA* Sequential Organ Failure Assessment

### Comparison of baseline characteristics among groups according to ICC and WIND classifications

Agreement of weaning results between ICC and WIND classifications is presented in Table 2. Although most patients in the simple weaning (462/503, 91.8%) or prolonged weaning groups (76/87, 87.4%) were classified as Group 1 or 3, respectively, only 59.3% (86/145) of patients in the difficult weaning group by ICC classification were classified as Group 2 by WIND classification. Of 865 patients whose weaning results could not be classified by ICC, 285 were classifiable to Group 1 (*n* = 109), 2 (*n* = 68), or 3 (*n* = 108) by WIND.

**Table 2:** Agreement according to ICC and WIND classifications

ICC	Simple	Difficult	Prolonged	Not classified	Total	Agreement, %
WIND						
Group NW	0	0	0	580	580	NA
Group 1	462	46	0	109	617	74.9
Group 2	21	86	11	68	186	46.2
Group 3	20	13	76	108	217	35.0
Total	503	145	87	865	1600	
Agreement, %	91.8	59.3	87.4	NA		39.0

For each line and column, agreement was calculated as follows: (number of patients classified in the same group by 2 classifications)/(total number of patients in line or column)

*ICC* International Consensus Conference, *WIND* Weaning according to a New Definition, *NW* no weaning, *NA* not accessible

In a comparison of baseline characteristics among weaning groups, there were statistically significant trends with more underlying malignancy and neurologic disorders, longer interval between hospital admission and ICU admission, more pneumonia as a cause of respiratory failure, less pulmonary edema as a cause of respiratory failure, and longer interval between hospital admission and intubation across the ICC classification from simple to prolonged weaning groups (Table 3). In addition to this trend, except for neurologic disorders, there were statistically significant trends with more respiratory disorders and less gastrointestinal and genitourinary disorders as underlying diseases across the WIND classification from Group 1 to Group 3.

**Table 3:** Comparison of baseline characteristics according to ICC and WIND classifications

Variables	ICC classification				WIND classification			
	Simple (*n* = 503)	Difficult (*n* = 145)	Prolonged (*n* = 87)	*P* for trend	Group 1 (*n* = 617)	Group 2 (*n* = 186)	Group 3 (*n* = 217)	*P* for trend
Age, years	65 (53–72)	68 (55–75)	67 (55–75)	0.081	65 (54–73)	66 (56–74)	66 (54–73)	0.774
Sex, male	351 (69.8)	93 (64.1)	64 (73.6)	0.952	427 (69.2)	127 (68.3)	147 (67.7)	0.673
Underlying disease								
Malignancy	249 (49.5)	81 (55.9)	61 (70.1)	< 0.001	296 (48.0)	116 (62.4)	151 (69.6)	< 0.001
Respiratory	135 (26.8)	42 (29.0)	31 (35.6)	0.106	163 (26.4)	57 (30.6)	82 (37.8)	0.002
Neurologic	62 (12.3)	24 (16.6)	17 (19.5)	0.042	94 (15.2)	35 (18.8)	36 (16.6)	0.484
Gastrointestinal	54 (10.7)	17 (11.7)	3 (3.4)	0.108	68 (11.0)	20 (10.8)	13 (6.0)	0.048
Cardiovascular	76 (15.1)	17 (11.7)	10 (11.5)	0.239	92 (14.9)	21 (11.3)	24 (11.1)	0.108
Genitourinary	74 (14.7)	17 (11.7)	10 (11.5)	0.292	90 (14.6)	12 (6.5)	21 (9.7)	0.014
Interval between hospital admission and ICU admission, days	1 (0–6)	1 (0–7)	3 (1–11)	0.001	1 (0–7)	2 (0–8)	3 (1–14)	< 0.001
Cause of respiratory failure								
Pneumonia	170 (33.8)	49 (33.8)	46 (52.9)	0.004	211 (34.2)	62 (33.3)	97 (44.7)	0.013
Extrapulmonary sepsis	104 (20.7)	30 (20.7)	10 (11.5)	0.095	111 (18.0)	38 (20.4)	31 (14.3)	0.343
ARDS	40 (8.0)	15 (10.3)	9 (10.3)	0.326	50 (8.1)	14 (7.5)	27 (12.4)	0.087
CPR	31 (6.2)	8 (5.5)	2 (2.3)	0.204	39 (6.3)	11 (5.9)	9 (4.1)	0.259
Coma	23 (4.6)	8 (5.5)	6 (6.9)	0.396	35 (5.7)	10 (5.4)	14 (6.5)	0.725
Pulmonary edema	45 (8.9)	10 (6.9)	1 (1.1)	0.014	48 (7.8)	9 (4.8)	4 (1.8)	0.001
Ventilatory failure	28 (5.6)	9 (6.2)	2 (2.3)	0.409	34 (5.5)	11 (5.9)	6 (2.8)	0.159
Central airway obstruction	14 (2.8)	2 (1.4)	3 (3.4)	1.000	30 (4.9)	9 (4.8)	5 (2.3)	0.143
Exacerbation of ILD	12 (2.4)	3 (2.1)	2 (2.3)	1.000	13 (2.1)	4 (2.2)	4 (1.8)	0.894
Others	36 (7.2)	11 (7.6)	6 (6.9)	1.000	46 (7.5)	18 (9.7)	20 (9.2)	0.332
SAPS III on ICU admission	61 (50–73)	63 (54–76)	64 (53–77)	0.046	61 (50–73)	63 (54–76)	65 (55–76)	0.001
SOFA score on ICU admission	8 (5–11)	8 (5–11)	8 (5–11)	0.514	8 (5–11)	8 (5–11)	9 (5–12)	0.063
Interval between hospital admission and intubation, days	1 (0–8)	2 (0–8)	3 (1–14)	< 0.001	1 (0–8)	3 (1–10)	4 (1–16)	< 0.001

*ICC* International Consensus Conference, *WIND* Weaning according to a New Definition, *ICU* intensive care unit, *ARDS* acute respiratory distress syndrome, *ILD* interstitial lung disease, *CPR* cardiopulmonary resuscitation, *SAPS III* Simplified Acute Physiology Score III, *SOFA* Sequential Organ Failure Assessment

### Clinical outcomes among groups according to ICC and WIND classifications

Clinical outcomes of the total cohort are listed in Table 4. Median interval between intubation and first SA was 3 days (IQR, 2–6 days), and median MV requirement was 5 days (IQR 2–11 days). Tracheostomy was needed in 416/1580 (26.3%) patients after a median of 11 days (IQR, 6–15 days) of intubation. The successful weaning rate was 51.5%, and ICU and hospital mortality were 41.0% and 53.0%, respectively.

**Table 4:** Clinical outcomes of total cohort, excluded patients by ICC classification, and Group NW by WIND classification

Variables	Total (*n* = 1600)	ICC	WIND
		Excluded (*n* = 865)	Group NW (*n* = 580)
Interval between intubation and the first SA, days^a^	3 (2–6)	4 (2–10)	–
SOFA score at the day of first SA^a^	5 (3–8)	7 (5–9)	–
MV days	5 (2–11)	6 (2–15)	5 (2–11)
Ventilator-free days^b^	2 (0–24)	0 (0–0)	0
Tracheostomy	436 (27.3)	277 (32.0)	101 (17.4)
No	1164 (72.8)	568 (65.7)	479 (82.6)
Before MV	20 (1.3)	20 (2.3)	6 (1.0)
Between MV and first SA	219 (13.7)	219 (25.3)	95 (16.4)
Between first SA and extubation	151 (9.4)	54 (6.2)	–
After the first extubation	46 (2.9)	4 (0.5)	–
Interval between intubation and tracheostomy, days^c^	11 (6–15)	9 (5–14)	10 (7–14)
Successful weaning from MV^d^	824 (51.5)	160 (18.5)	0
ICU mortality	656 (41.0)	588 (68.0)	511 (88.1)
LOS in ICU, days	7 (4–15)	8 (3–17)	6 (2–13)
Hospital mortality	848 (53.0)	643 (74.3)	520 (89.7)
LOS in hospital, days	24 (13–46)	20 (9–43)	15 (5–30)
Type of discharge			
Home	422 (26.4)	44 (5.1)	0
Other hospital	219 (13.7)	90 (10.4)	5 (0.9)
Other ICU	24 (1.5)	20 (2.3)	10 (1.7)
Hospice	87 (5.4)	68 (7.9)	45 (7.8)
Death	848 (53.0)	643 (74.3)	520 (89.7)

*ICC* International Consensus Conference, *NW* no weaning, *WIND* Weaning according to a New Definition, *SA* separation attempt, *SOFA* Sequential Organ Failure Assessment, *MV* mechanical ventilation, *ICU* intensive care unit, *LOS* length of stay

^a^Excluded patients who had no SA from MV. Therefore, total patients, excluded patients by ICC, and Group NW by WIND numbered 1020, 285, and 0, respectively

^b^Ventilator-free days are defined as 28 minus the total number of days with invasive MV. Nonsurvivors were considered as having 0 ventilator-free days

^c^Excluded patients with no tracheostomy or tracheostomy prior to mechanical ventilation

^d^Successful weaning is defined as in the WIND Study (Intubated patients: extubation without death or reintubation within 7 days after extubation [whether postextubation noninvasive ventilation was used or not] or ICU discharge without invasive mechanical ventilation within 7 days, whichever comes first. Tracheostomized patients: spontaneous ventilation through tracheostomy without any mechanical ventilation during 7 consecutive days or ICU discharge with spontaneous breathing, whichever comes first)

**Fig. 2:**
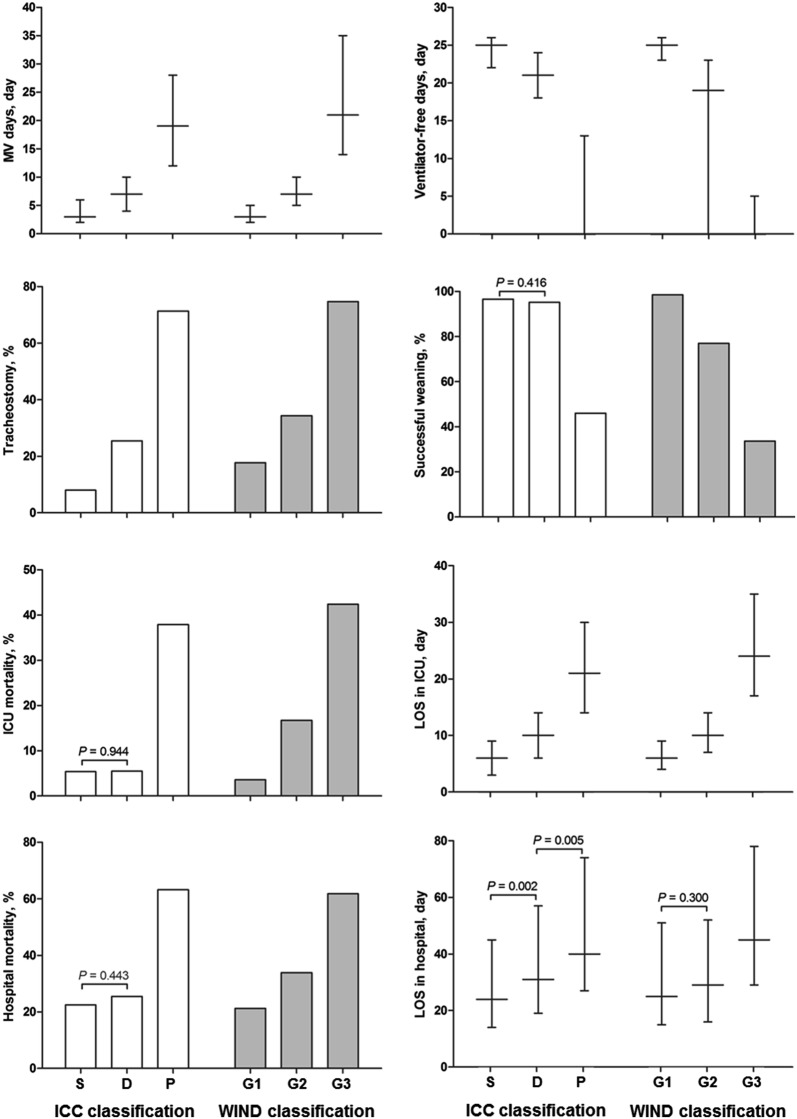
Comparisons of weaning outcomes between groups according to ICC and WIND classifications. Data are presented as medians and interquartile ranges for continuous variables and as percentages for categorical variables. *P* values between groups are < 0.001 except where otherwise noted. *ICC* International Consensus Conference, *WIND* Weaning according to a New Definition, *MV* mechanical ventilation, *ICU* intensive care unit, *LOS* length of stay, *G1* Group 1, *G2* Group 2, *G3* Group 3, *S* simple weaning group, *D* difficult weaning group, *P* prolonged weaning group

All of these clinical outcomes showed statistically significant trends across the ICC and WIND classifications (Table 5). However, there were no statistical differences in successful weaning rate (96.6% vs. 95.2%, *P* = 0.416), ICU mortality (5.4% vs. 5.5%, *P* = 0.944), and hospital mortality (22.5% vs. 25.5%, *P* = 0.443) between simple and difficult weaning groups by ICC (Fig. 2). Conversely, there were statistically significant differences in successful weaning rate (98.5% vs. 76.9%, *P* < 0.001), ICU mortality (3.6% vs. 16.7%, *P* < 0.001), and hospital mortality (21.2% vs. 33.9%, *P* < 0.001) between Group 1 and Group 2 by WIND. By the WIND classification, only the LOS between Group 1 and Group 2 had no statistically significant difference (median 25 days [IQR 15–51 days] versus median 29 days [IQR 16–52 days], *P* = 0.300).

**Table 5:** Clinical outcomes according to ICC and WIND classifications

Variables	ICC classification				WIND classification			
	Simple (*n* = 503)	Difficult (*n* = 145)	Prolonged (*n* = 87)	*P* for trend	Group 1 (*n* = 617)	Group 2 (*n* = 186)	Group 3 (*n* = 217)	*P* for trend
Interval between intubation and the first SA, days	3 (2–5)	3 (2–6)	4 (2–7)	0.002	3 (2–5)	3 (2–6)	4 (2–9)	< 0.001
SOFA score at the day of first SA	5 (3–7)	6 (3–8)	5 (4–8)	< 0.001	5 (3–7)	6 (4–8)	7 (5–9)	< 0.001
MV days	3 (2–6)	7 (4–10)	19 (12–28)	< 0.001	3 (2–5)	7 (5–10)	21 (14–35)	< 0.001
Ventilator-free days^a^	25 (22–26)	21 (18–24)	0 (0–13)	< 0.001	25 (23–26)	19 (0–23)	0 (0–5)	< 0.001
Tracheostomy	40 (8.0)	37 (25.5)	62 (71.3)	< 0.001	109 (17.7)	64 (34.4)	162 (74.7)	< 0.001
No	463 (92.0)	108 (74.5)	25 (28.7)	–	508 (82.3)	122 (65.6)	55 (25.3)	–
Before MV	–	–	–	–	10 (1.6)	3 (1.6)	1 (0.5)	–
Between MV and first SA	–	–	–	–	63 (10.2)	21 (11.3)	40 (18.4)	–
Between first SA and extubation	12 (2.4)	25 (17.2)	60 (69.0)	–	0	32 (17.2)	119 (54.8)	–
After the first extubation	28 (5.6)	12 (8.3)	2 (2.3)	–	36 (5.8)	8 (4.3)	2 (0.9)	–
Interval between intubation and tracheostomy, days^b^	18 (13–33)	12 (5–19)	11 (9–15)	< 0.001	10 (2–17)	7 (4–12)	12 (9–15)	< 0.001
Successful weaning from MV^c^	486 (96.6)	138 (95.2)	40 (46.0)	< 0.001	608 (98.5)	143 (76.9)	73 (33.6)	< 0.001
ICU mortality	27 (5.4)	8 (5.5)	33 (37.9)	< 0.001	22 (3.6)	31 (16.7)	92 (42.4)	< 0.001
LOS in ICU, days	6 (3–9)	10 (6–14)	21 (14–30)	< 0.001	6 (4–9)	10 (7–14)	24 (17–35)	< 0.001
Hospital mortality	113 (22.5)	37 (25.5)	55 (63.2)	< 0.001	131 (21.2)	63 (33.9)	134 (61.8)	< 0.001
LOS in hospital, days	24 (14–45)	31 (19–57)	40 (27–74)	< 0.001	25 (15–51)	29 (16–52)	45 (29–78)	< 0.001
Type of discharge								
Home	296 (58.8)	72 (49.7)	10 (11.5)	–	343 (55.6)	62 (33.3)	17 (7.8)	–
Other hospital	82 (16.3)	30 (20.7)	17 (19.5)	–	129 (20.9)	45 (24.2)	40 (18.4)	–
Other ICU	0	1 (0.7)	3 (3.4)	–	0	4 (2.2)	10 (4.6)	–
Hospice	12 (2.4)	5 (3.4)	2 (2.3)	–	14 (2.3)	12 (6.5)	16 (7.4)	–
Death	113 (22.5)	37 (25.5)	55 (63.2)	–	131 (21.2)	63 (33.9)	134 (61.8)	–

*ICC* International Consensus Conference, *WIND* Weaning according to a New Definition, *SA* separation attempt, *SOFA* Sequential Organ Failure Assessment, *MV* mechanical ventilation, *ICU* intensive care unit, *LOS* length of stay

^a^Ventilator-free days are defined as 28 minus the total number of days with invasive MV. Nonsurvivors were considered as having 0 ventilator-free days

^b^Excluded patients with no tracheostomy and tracheostomy prior to mechanical ventilation

^c^Successful weaning is defined as in the WIND Study (Intubated patients: extubation without death or reintubation within 7 days after extubation [whether postextubation noninvasive ventilation was used or not] or ICU discharge without invasive mechanical ventilation within 7 days, whichever comes first. Tracheostomized patients: spontaneous ventilation through tracheostomy without any mechanical ventilation during 7 consecutive days or ICU discharge with spontaneous breathing, whichever comes first)

## Discussion

To the best of our knowledge, this is the first validation study of WIND classification compared to ICC classification. Our study demonstrates that the WIND classification could be operational for every patient under MV and better discriminates clinical outcomes by weaning group compared to ICC classification.

In this study, only 46% of patients receiving invasive MV were classifiable by ICC. However, WIND classification was applicable to all patients, even in tracheostomized patients and patients not receiving the SBT. Our results are similar to those of the original study that proposed WIND classification, which classified only 1330/2709 (51%) patients by ICC and all patients by WIND [8]. In previous studies related to ICC classification, 40–60% of mechanically ventilated patients were excluded from studies because they died, had a tracheostomy, transferred to another hospital, had unplanned extubation before they were ready to wean or during weaning, or did not use SBT to wean [3, 4, 6, 7]. However, all patients could adopt the WIND classification because (a) the starting point of weaning in WIND classification was defined as SA including methods other than SBT, even unplanned extubation, (b) WIND classification provided clear criteria for the starting point of weaning and successful weaning in both intubated and tracheostomized patients, and (c) the WIND classification is based on duration of ventilation between the first SA and the end of weaning, regardless of the results, such as successful separation or death.

Although most previous studies have shown that prolonged weaning increases ICU and hospital mortality rates, there are no statistical differences between simple and difficult weaning [3, 4, 6, 7, 13]. As with previous studies, ICU and hospital mortality and successful weaning rates between simple and difficult weaning groups by ICC classification showed no differences in the present study. However, WIND classification had stepwise differences in Groups 1–3 for these weaning outcomes. In Table 4, successful weaning was noted in 18.5% (160/865) of the unclassifiable patients by ICC. In addition, their ICU survival rate was 32.0%, which was higher than that of Group NW (11.9%). Because these patients were classified as Groups 1–3 by WIND, the WIND classification seems to show greater differences in weaning outcomes between groups than does the ICC classification.

Although this study provides new information on weaning outcome based on new definitions that allow classification of all mechanically ventilated patients, our study has some limitations that should be considered. First, given its observational nature in a single tertiary referral hospital, there could be a selection bias that might have influenced the significance of our results. However, the data were collected prospectively between July 2010 and September 2013 from all consecutive patients who were admitted to the medical ICU and mechanically ventilated for more than 24 h. The patients were screened daily for weaning readiness according to a protocol-based weaning program [3, 9]. Thus, our cohort is more likely to reflect the patients encountered in routine ICU practice, and our findings are therefore readily applicable in similar settings. Second, our cohort was weaned from MV according to a protocol-based program with SBT using a T-piece. In addition, tracheostomy was performed in a quarter of patients, which is higher than the rate of 11–15% in an international multicenter study [14]. Although SBT using a T-piece is a general method of withdrawal from MV [4] and tracheostomy may improve aspects of care of patients on MV [15], our findings have limitations in their generalizability to other groups that underwent methods such as SBT using low pressure support, continuous positive airway pressure, gradual reduction in support using pressure support mode, or synchronized intermittent mandatory ventilation, and that has lower rate of tracheostomy.

## Conclusion

In conclusion, WIND classification could be a better tool for predicting weaning outcomes than ICC classification because WIND classification is applicable to all mechanically ventilated patients and has higher discriminatory power for weaning outcomes.

## Additional file

**Additional file 1.** Standardized weaning process.

## Abbreviations

ICC: International Consensus Conference
ICU: intensive care unit
IQR: interquartile range
LOS: length of stay
MV: mechanical ventilation
NIV: noninvasive ventilation
NW: no weaning
RCP: respiratory care practitioners
REVA: Réseau Européen de Recherche en Ventilation Artificielle
SA: separation attempt
SBT: spontaneous breathing trial
WIND: Weaning according to a New Definition
