# Supplementary material for: Validation of a new WIND classification compared to ICC classification for weaning outcome
Source: Ann Intensive Care. 2018 Nov 29;8:115. doi: 10.1186/s13613-018-0461-z (PMC6265356; doi:10.1186/s13613-018-0461-z)
Supplement: Supplementary file 1 — Additional file 1. Standardized weaning process. [file 13613_2018_461_MOESM1_ESM.docx]

**Additional file. Standardized weaning process**

A specific protocol-based weaning program based on the then recommendations [[1](#_ENREF_1)] has been implemented in our center since 2010 (Figure S1). Respiratory care practitioners (RCPs), registered nurses specializing in respiratory care, screened patients daily for weaning readiness according to the criteria adapted from the recommendations [[1](#_ENREF_1)] (Table S1). The weaning protocol was initiated when the Richimond Agitation-Sedation Scale score was 0 to -1 after sedation vacation in the morning. The patients who fulfilled these criteria were assessed for the likelihood of a successful spontaneous breathing trial (SBT) with a calculation of the rapid shallow breathing index (RSBI, the ratio of respiratory frequency to tidal volume) on continuous positive airway pressure (CPAP) of 5 cmH_2_O for 3 minutes [[2](#_ENREF_2)]. If the RSBI was less than 105, the patients underwent a SBT as a diagnostic test to assess the likelihood of successful weaning. The initial SBT consisted of breathing with a T-piece at 9–10 L/min with 40% inspiratory oxygen fraction (FiO_2_) and lasted 30 min. When a patient successfully passed the SBT according to the criteria (Table S2), the patient underwent the cuff-leak test for extubation readiness as described by Krinner *et al* [[3](#_ENREF_3)]. When a patient failed the cuff-leak test (absolute volume < 110 mL or 15% of exhaled volume), the patient received intravenous infusion of methylprednisolone every 6 hours over 24 hours before extubation [[4](#_ENREF_4)] and then was extubated without repeated cuff-leak test. When a patient successfully passed the cuff-leak test, extubation proceeded immediately. If a patient failed the SBT, MV was restarted, and the team reviewed the possible reversible etiologies for the failure. When the patient was again ready for weaning, the SBT was repeated for 120 min on the next day.

**References**

1. Boles JM, Bion J, Connors A, Herridge M, Marsh B, Melot C, et al. Weaning from mechanical ventilation. Eur Respir J. 2007;29:1033-56.

2. Patel KN, Ganatra KD, Bates JH, Young MP. Variation in the rapid shallow breathing index associated with common measurement techniques and conditions. Respir Care. 2009;54:1462-6.

3. Kriner EJ, Shafazand S, Colice GL. The endotracheal tube cuff-leak test as a predictor for postextubation stridor. Respir Care. 2005;50:1632-8.

4. Cheng KC, Hou CC, Huang HC, Lin SC, Zhang H. Intravenous injection of methylprednisolone reduces the incidence of postextubation stridor in intensive care unit patients. Crit Care Med. 2006;34:1345-50.

**Figure S1**. Weaning protocol. SBT = spontaneous breath trial, CPAP = continuous positive airway pressure, RSBI = rapid shallow breathing index (respiratory rate [in breaths/min] / tidal volume [in liters]).


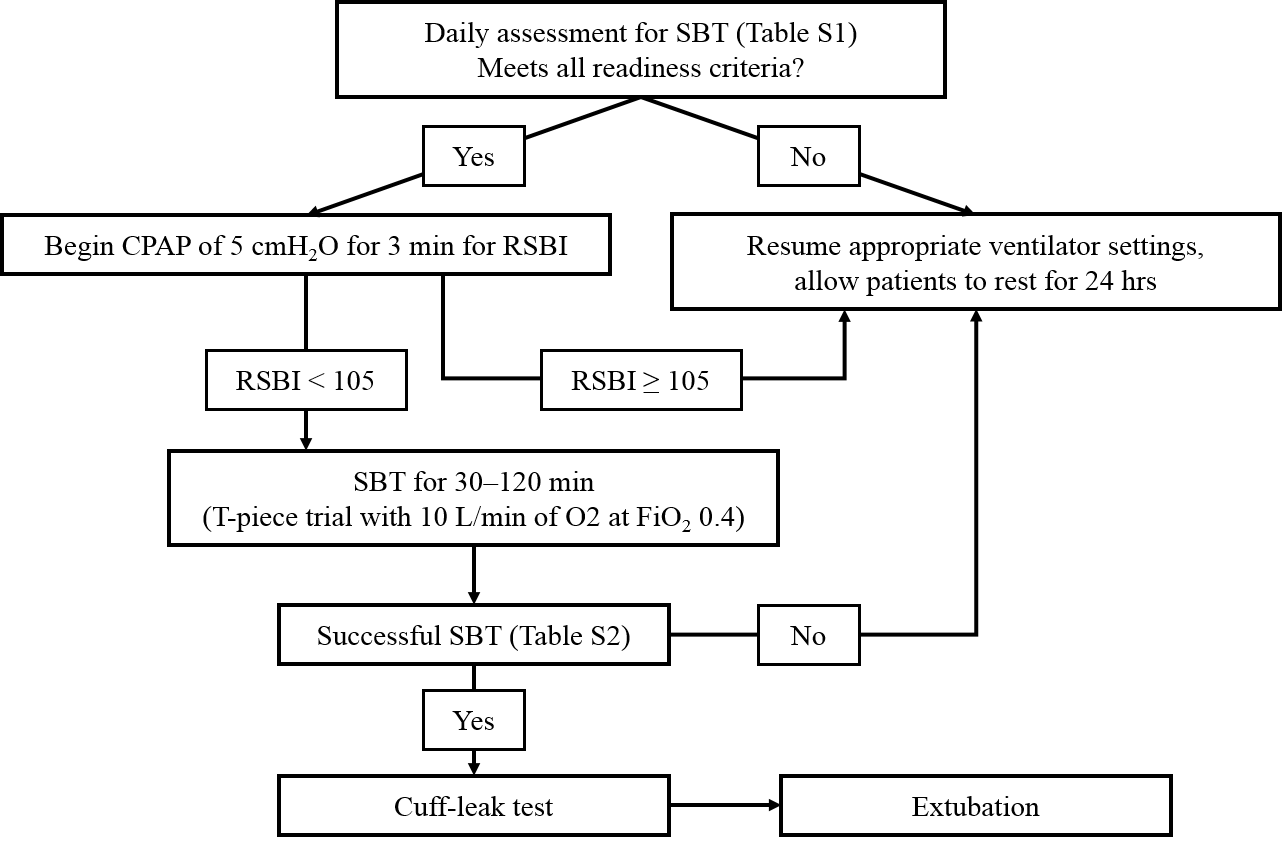


**Table S1**. Considerations in daily assessing readiness for weaning.

| Clinical assessment | Adequate cough  Absence of excessive tracheobronchial secretion  Resolution of disease acute phase for which the patient was intubated |
| --- | --- |
| Objective measurements | *Clinical stability*  Stable cardiovascular status (i.e. HR ≤ 120/min, SBP 90~140 mmHg, no or minimal vasopressors: dopamine ≤ 5 mcg/kg/min, norepinephrine ≤ 0.05 mcg/kg/min)  Stable metabolic status  *Adequate oxygenation*  SaO_2_ > 90% on FiO_2_ ≤ 0.4 (or PaO_2_/FiO_2_ ratio > 200)  PEEP ≤ 5 cmH_2_O  *Adequate pulmonary function*  RR < 35/min  Negative inspired pressure (NIP)^*^ ≤ -15 cmH_2_O  V_T_ > 5 mL/kg  V_E_ < 10~15 L/min  No significant respiratory acidosis  *Adequate mentation*  No sedation or adequate mentation on sedation |

HR = heart rate, SBP = systolic blood pressure, SaO_2_ = arterial oxygen saturation, FiO_2_ = inspiratory oxygen fraction, PaO_2_ = arterial oxygen tension, PEEP = positive end expiratory pressure, RR = respiratory rate, V_T_ = tidal volume, V_E_ = minute ventilation.

^*^ Negative inspiratory pressure (NIP) is the lowest pressure generated during a forceful inspiratory effort against an occluded airway, which is determined by occluding the ventilator’s inspiratory port at the end of expiration for 20 seconds and reading the maximum negative pressure registered on the ventilator’s pressure manometer.

**Table S2**. Criteria for SBT failure.

| Clinical assessment | Agitation and anxiety  Depressed mental status  Diaphoresis  Cyanosis  Evidence of increasing effort  Increased accessory muscle activity  Facial signs of distress  Dyspnea |
| --- | --- |
| Objective measurements | PaO_2_ < 60 mmHg or SaO_2_ < 90% on FiO_2_ ≥ 0.4  PaCO_2_ > 45 mmHg or an increase in ≥ 20% from pre-SBT and pH < 7.32 and a decrease in pH ≥ 0.07  RR > 35/min or increased by ≥ 50%  HR > 140/min or increased by ≥ 20%  SBP > 180 mmHg or increased by ≥ 20%  SBP < 90 mmHg  Cardiac arrhythmias |

SBT = spontaneous breathing trial, PaO_2_ = arterial oxygen tension, SaO_2_ = arterial oxygen saturation, FiO_2_ = inspiratory oxygen fraction, PaCO_2_ = arterial carbon dioxide tension, RR = respiration rate, HR = heart rate, SBP = systolic blood pressure.
